# Nanofiber embedded bioinspired strong wet friction surface

**DOI:** 10.1126/sciadv.adi4843

**Published:** 2023-10-12

**Authors:** Yurun Guo, Liwen Zhang, Yan Wang, Jing Liang, Xiaolin Liu, Yonggang Jiang, Lei Jiang, Huawei Chen

**Affiliations:** ^1^ School of Mechanical Engineering and Automation, Beihang University, Beijing, China.; ^2^ Laboratory of Bioinspired Smart Interface Science, Technical Institute of Physics and Chemistry, Chinese Academy of Sciences, Beijing, China.; ^3^ Beijing Advanced Innovation Centre for Biomedical Engineering, Beihang University, Beijing, China.

## Abstract

Robust and reversible wet attachments are important for medical engineering and wearable electronics. Although ultrastrong capillarity from interfacial nano-thick liquid bridges creates tree frog’s strong wet friction, its unstable nano-liquid characteristic challenges further wet friction enhancement. Here, unique hierarchical micro-nano fibrous pillars have been discovered on Chinese bush crickets exhibiting a robust wet friction ~3.8 times higher than tree frog’s bulk pillar. By introducing a nano-fibrous pillar array covered with thin films (NFPF), the pillar’s separation position switches from the rear to front side compared with bulk pillars, indicating the interfacial contact stress shifting from compressing to stretching. This largely decreases the interfacial separation stress to form more stable and larger nano-liquid bridges. The NFPF array with self-splitting of interfacial liquid and contact stress further guards such interfacial stress shifting to ensure a ~1.9 times friction enhancement. Last, the theories are established, and the applications on wearable electronics are validated.

## Teaser

Nanofiber embedded micropillar surfaces with strong wet friction from the interfacial contact stress self-splitting effect.

## INTRODUCTION

Wet attachment widely exists in diverse biomechanical contact interfaces, such as surgical graspers and artificial limbs. In particular, with the rapid progress of precision medicine, the robust contact between wearable flexible electronics and human tissues or organs has aroused considerable interest over the past several decades (*1*–*6*). However, because of the existed liquid film between the contact interfaces, the devices could easily slip off from the biological surface, resulting in serious consequences including tissue damage or sensor detection failure (*7*–*12*). Developing functional surfaces with exceptional wet attachment properties on slippery skins is in immense demand to avert undesirable slippage, which will be advantageous to its potential applications in medical engineering and wearable flexible electronics.

To date, two strategies have been mainly introduced to generate strong wet adhesion, including soft polymer materials to create molecular bonds on the surface (*13*–*17*) or soft suckers to produce vacuum pressure by external atmospheric pressure (*18*–*22*). However, these adhesive strategies are always hampered by either low reusability of the irreversible interfacial chemical bonds or the requirement of thorough sealing and external preloading force, which both limit their applications on soft and nonflat skin contact treatments. Although reversible hydrogel tissue adhesives have been reported over the past years, the detachment process may cause damage to tissues or require external stimulus (*23*–*29*). Luckily, the extraordinary attachment properties of natural creatures evolving over millions of years have enlightened the design and fabrication of robust and reversible wet adhesion (*30*–*33*). Tree frog with its unique hierarchical micro-nano pillar has been discovered with superb interfacial liquid adjusting ability to create a great number of uniform nano-thick liquid bridges between toe pad and substrate. These liquid bridges are demonstrated to automatically form an ultrastrong capillarity and produce robust wet boundary friction with advantages of reusability and needless external normal pressure (*34*–*36*). Such nano-liquid film wet adhesive mechanism shines a promising wet adhesive approach for improved adaptivity in medical and wearable devices. However, these nano-interfacial liquid bridges could be easily broken during sliding due to the deformation of soft pillars and the movement of nano liquid film, accompanied by the sudden disappearance of boundary friction. How to stabilize the nano-thick interfacial liquid film even under the deformation of the soft surface structure becomes critical for further enhancement of wet friction.

Soft micro-nano surface structures as intrinsic properties of natural creatures can create varied structural deformation and greatly alter the distribution and transmission of interfacial contact stress, which eventually determines the surface’s contact force performance. For example, pillar array covered with a large thin membrane forms nonuniform surface elasticity and deformation to discontinue interfacial cracks during detachment and generate higher adhesion (*37*). In addition, composite surface structures with rigid cores embedded in soft shells could shift the maximum stress toward the central region to enhance the adhesion (*38*, *39*). However, with the presence of interfacial liquid, the coupling effect from soft deformation and stress transmission on liquid’s fluidity and capillarity has barely been discussed because of the difficulties of in situ micro-nano interfacial characterization. The mutual dynamic interactions between the soft structure deformation, interfacial liquid flow, and strong capillarity from nano liquid bridges still need to be revealed for the enhancement of wet friction.

In this work, the strong wet attachment pads of the Chinese bush crickets are discovered with unique hierarchical micro-nano structures, where each micropillar is embedded with long nanofibers and covered by a smooth thin film. Under such inspiration, NFPF has been designed and fabricated by integrated processes of anodic aluminum oxide (AAO) mold replication and photolithography. With the in situ observation of interfacial liquid movement and pillar deformation, an exceptional interfacial stress shifting effect along the unique hierarchical structured surface has been found and the interfacial nano-liquid film has been maintained in a more stable state, thus creating much stronger wet boundary friction. By applying such strong surfaces on several wearable sensors, its strong wet attachment performance has been validated and offers potential application in the fields of medicine and mechanical engineering.

## RESULTS

### Structural characteristics and friction performance of the Chinese bush cricket attachment pads

**Fig. 1.**
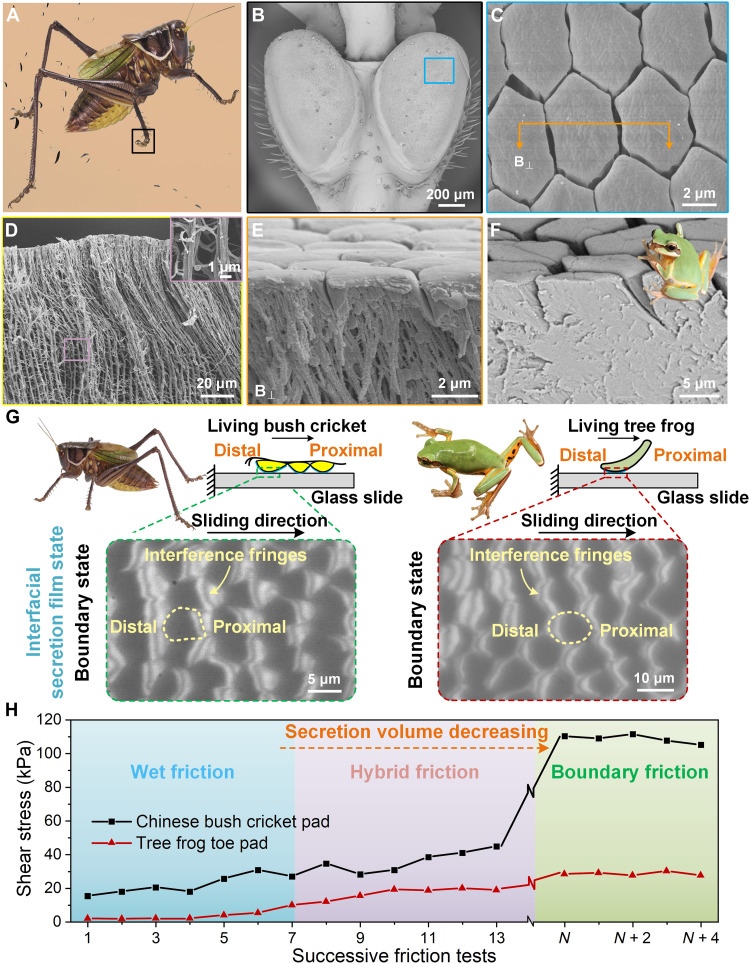
Structure and friction performance of the attachment pads of the Chinese bush cricket (*G. gratiosa*, *Tettigoniidae*) and tree frog (*Polypedates and Rhacophorus dennysi*). (**A**) A photo of a bush cricket crawling on a slippery glass. (**B** and **C**) One pair of attachment pads with surface composed of tightly packed hexagonal epithelial cells had a diameter, height, and channel width of about 5, 2, and 0.2 μm, respectively. Magnified epithelial cells show a smooth outer layer. (**D**) Section view of the pad after critical point drying shows that the branched fibrous structures extend from ~100-μm depth in the cuticle to the top surface of the hexagonal pillar. The inset shows that the diameter of the root fiber is ~1 μm. (**E**) The hexagonal pillars are embedded inside with tiny fibers that are dispersed from the root fibers and have a diameter of ~100 nm. (**F**) Section view of the epithelial cell from the tree frog’s toe pad shows homogeneous pillar structures. (**G**) The interfacial secretion film states of cricket’s and tree frog’s pads during repeated sliding process. The dark areas denote the interfacial secretion film at contact interface. The yellow dashed circles indicate the individual epithelial cells, and the yellow arrows indicate the positions where interference fringes appear, representing the separation of cells and glass substrate. (**H**) The shear stress of cricket’s and tree frog’s pads during repeated sliding process. All friction tests are measured with a normal load of ~1 kPa and a contact area of ~1 mm^2^.

The Chinese bush cricket (*Gampsocleis gratiosa*, *Tettigoniidae*) can freely climb or even hang upside down on wet smooth surfaces to prey or avoid attacks, exhibiting an extraordinary wet attachment performance (Fig. 1A). Its attachment pad’s surface is covered by tightly packed hexagonal pillars with diameter, height, and channel width of about 5, 2, and 0.2 μm, respectively, which is similar to the morphology of tree frog’s toe pad except on a smaller scale (Fig. 1, B and C). Different from the tree frog with solid bulk pillar (BP), the cricket’s pillar is embedded with long and thin nanofiber branches, which root deeply into the cuticle with a diameter of ~1 μm and extend to the top smooth pillar surface with a diameter of ~100 nm (Fig. 1, D to F). To further explore the distribution of tissue fluid in cricket’s pad, a frozen sample is characterized cross-sectionally by cryo–scanning electron microscopy and shows that the gaps between nanofiber arrays are fulfilled with tissue fluid (fig. S1). Such a unique complex hierarchical structure with liquid saturated could create an ultrasoft surface to form closer contact on rough substrates and change the contact stress transmission, and its wet friction performance could be quite different from the tree frog’s toe pad (fig. S2).

To investigate the effect of interfacial liquid on wet attachment, tests of successive step sliding have been carried out for the cricket and tree frog’s pads, where a glass substrate repeatedly slides from the pad’s proximal side to the distal side under a normal load of ~1 kPa to mimic their crawling steps (Fig. 1G and fig. S3A). The friction force and interfacial secretion film states are simultaneously monitored during each sliding step. With successive sliding, the interfacial secretion volume on cricket’s and tree frog’s pads gradually decreases and their friction increases, which eventually reaches a peak accompanied by the liquid films discretely and uniformly distribute on each pillar, i.e., the boundary friction state (Fig. 1G, bottom). The cricket exhibits much strong boundary friction to be ~100 times its normal force and ~3.8 times higher than the friction of the tree frog (Fig. 1H). In boundary friction state, interfacial in situ observation shows that interference fringes appear at each pillar top surface during sliding, indicating that these pillars are separated from the substrate with air entering the contact interfaces (movie S1). The interference fringes appear at each pillar’s proximal side on cricket while at the distal side on tree frog with opposite separating position (Fig. 1G, bottom). Such distinct interfacial liquid film dynamic behaviors could be the reason for the cricket’s outstanding friction performance. To eliminate the effects from attachment pad secretion and reveal the underlying mechanism, a detailed characterization needs to be carried out via bioinspired wet attachment surfaces.

### Friction performance of bioinspired surfaces

**Fig. 2.**
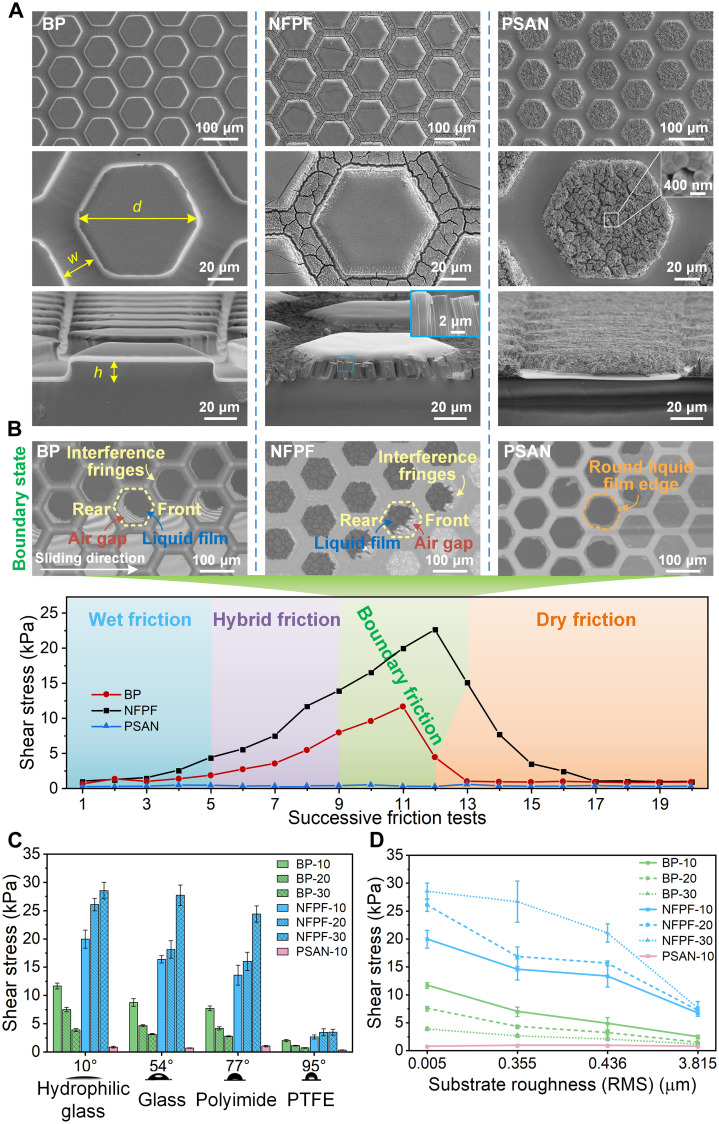
Characterization of structures and wet friction properties of BP, PSAN, and NFPF surfaces. (**A**) Scanning electron microscopy images of different types of pillars with the diameter *d*, channel width *w*, and height *h* of 100, 25, and 10 μm, respectively. Nanofibers in NFPF and PSAN with a diameter of 400 nm. (**B**) Shear stress for different surfaces during successive friction tests, and their interfacial liquid film states at boundary friction are compared. Interference fringes are only observed on BP and NFPF surfaces. The air gap appears at the rear side of BP and the front side of NFPF during sliding. (**C**) Boundary frictional shear stress for different pillar surfaces on various hydrophilic substrates. (**D**) Boundary frictional shear stress for different pillar surfaces on various roughness substrates. BP/NFPF-number denotes BP/NFPF of the specific height. All friction tests were conducted without external normal force and with an area of ~1 cm^2^. PTFE, polytetrafluoroethylene; RMS, root mean square. Values in (C) and (D) represent the mean, and the error bars represent SD of the measured values (*n* = 5).

Inspired from natural creatures, three types of bioinspired surfaces were designed and fabricated for revealing wet friction mechanism, including BP array from the tree frog, NFPF from the cricket, and hexagonal pillar array composed of self-assembled nanofibers (PSAN) (Fig. 2A and fig. S4). To avoid the cluster of nanofibers, the AAO templates used in the fabrication of NFPF and PSAN were predeposited with parylene coatings. After a volume of 1.5 μl of deionized water was injected onto the bioinspired surface, a glass substrate was put on the bioinspired surface without applying additional vertical force and moved horizontally to conduct the friction test. After completing a test, the glass substrate was lifted vertically and moved to a clean area to repeat the above test process until the water on the contact area disappeared (fig. S3B).

By applying the successive sliding tests on these surfaces, the PSAN exhibits almost no friction in all testing steps. Both BP and NFPF show similar friction variations as natural creatures’ pads until they eventually enter the dry state almost without friction, which is unlike the maintainable boundary friction on creatures by constantly secreted mucus (Fig. 2B, bottom). The normal adhesion of NFPF exhibits similar trend of the friction, i.e., increasing first and then decreasing to almost zero (fig. S5, A and B). The BP and NFPF surfaces exhibit comparable maximum normal adhesion stress, while the PSAN shows much lower adhesion (fig. S5C). The different wet friction performance of PSAN, BP, and NFPF surfaces could result from their different interfacial liquid film thicknesses. The liquid’s edge on the PSAN is smooth and round without any interference fringes, which indicates a relatively thick liquid film with small capillarity to form the low boundary friction (Fig. 2B, top right). This could be attributed to the high roughness of the PSAN surface caused by the collapse of nanofibers (fig. S6). During boundary friction sliding, the separating positions on BP and NFPF appear at the rear side and the front side, respectively, which is identical to the phenomenon observed on pads from tree frog and cricket (Fig. 2B, top, left, and middle). Such interfacial liquid states endow NFPF surfaces with much strong boundary friction to be ~1.9 times higher than BP surface (Fig. 2B, bottom).

Furthermore, more structural and material properties’ impacts on boundary friction have been characterized. With substrates contact angle increasing from 10° to 95°, the boundary friction decreases distinctly on all types of surfaces, resulting from the decline of interfacial capillarity (Fig. 2C). Similarly, with increasing contact angle of bioinspired surfaces, the boundary friction also decreases (fig. S7D). By increasing the pillar height from 10 to 30 μm, the BP surface’s boundary friction decreases about 64%, while the NFPF surface’s increases about 40%, leading to the NFPF-30’s boundary friction to be about 7.4 times of BP-30’s (fig. S8). However, further increasing the pillar height of NFPF surfaces does not enhance the boundary friction. The boundary friction of NFPF-50 is similar to that of NFPF-30 (fig. S9, A and B). The micro-nano hierarchical structure of NFPF with nanofiber height of 30 μm still has a high mechanical stability even after 20 times of friction tests (fig. S7E). With substrates roughness increasing from 0.005 to 3.815 μm, the boundary friction of all types of surfaces decreases, while the NFPF surface still maintains much higher friction than BP and PSAN surfaces (Fig. 2D). A similar situation also occurs in cricket pads, where the boundary friction gradually decreases as the substrate roughness increases (fig. S10). The bioinspired surfaces with higher elastic modulus show lower boundary friction properties (fig. S7, A to C). In addition, bioinspired NFPF surfaces with smaller pillar diameter could maintain stronger boundary friction on rough surfaces (fig. S11). This could result from that it is more difficult for larger pillars to maintain nano-thick liquid film on a rough substrate. Such exceptional wet friction performance on NFPF and its corresponding interfacial liquid state could result from the unique hierarchical micro-nano fibrous pillar structure, and the underlying mechanism needs more detailed characterization.

### Interfacial liquid film and pillar dynamic behaviors on bioinspired surfaces

**Fig. 3.**
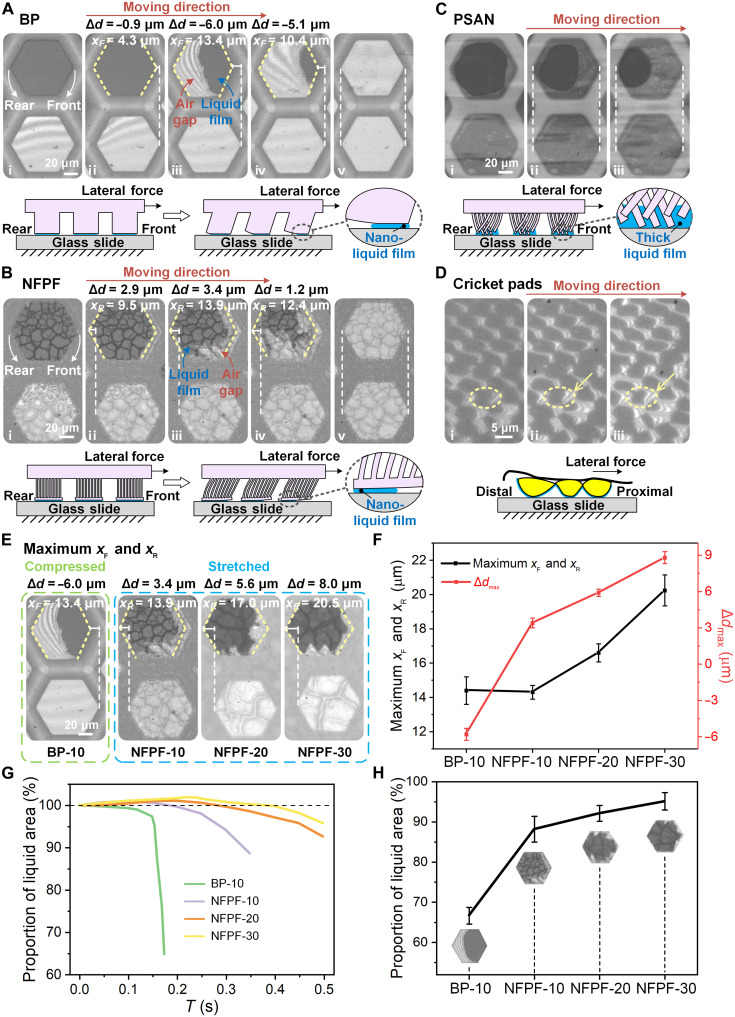
Characterization of interfacial liquid film behavior and structural deformation of different pillars in the boundary friction process. (**A** to **C**) The interfacial liquid film states and pillar deformation of the BP, NFPF, and PSAN during the sliding in boundary friction. By comparing the adjacent dry and boundary state pillars, the pillar deformation and the interfacial acting force can be achieved. The yellow dashed lines mark the pillar front and rear borders. *x*_F_ and *x*_R_ represent the deformation at the front and rear sides of pillars in boundary friction. ∆*d* denotes the change in pillar diameter. The BP and NFPF initially adhere to substrate by capillarity from interfacial nano-thick liquid film and then start to deform and separate from substrate under the shearing action. The separating positions of BP and NFPF are at the rear and front sides of the pillar, respectively. For PSAN, liquid film with smooth round edge and dim color indicates a thick liquid film, and no clear deformation of the pillar occurs under sliding. (**D**) The interfacial secretion film state of the cricket pad sliding from distal to proximal direction on a glass substrate. The yellow circles denote individual pillars attaching to the glass substrate, and yellow arrows indicate the interference fringes formed between pillars and substrate. Each schematic diagram at the bottom of (A) to (D) corresponds to its friction process. (**E** and **F**) The maximum *x*_F_, *x*_R_, and Δ*d* of BP, NFPF-10, NFPF-20, and NFPF-30 in boundary friction with BP being compressed and NFPF being stretched. (**G**) The interfacial liquid film area changes on different pillars during boundary friction. (**H**) The interfacial liquid film area proportions on different pillars before utter separation at maximum displacement. Values in (F) and (H) represent the mean, and the error bars represent SD of the measured values (*n* = 5).

The interfacial liquid film and structural dynamic behaviors were in situ characterized on bioinspired surfaces during boundary friction via a microscope with high-speed camera. On BP and NFPF surfaces with lateral sliding force applied, the pillars initially adhere to substrate by capillarity from interfacial nano-thick liquid film and then start to deform and separate from substrate under the shearing action from friction and lateral sliding force (Fig. 3, A and B). On BP, a clear extruding force appears on pillar surface with pillar surface separating from the rear side, resulting in a maximum lateral displacement *x*_F_ = 13.4 μm and a shorter pillar with a diameter change ∆*d* of −6.0 μm (Fig. 3A; movie S2; and fig. S12, A and C). Oppositely, a distinct stretching deformation is formed on NFPF pillar accompanied by pillar top surface separating at the front side, leading to a maximum *x*_R_ = 13.9 μm and a longer pillar with ∆*d* of 3.4 μm (Fig. 3B; movie S3; and fig. S12, B and C). Such front side pillar separation and diameter increase can also be observed on cricket pad, suggesting a similar interfacial force acting behavior between NFPF and cricket pad (Fig. 3D). For PSAN, a much thicker liquid film forms due to its rough top pillar surface and results in much weaker capillarity. The thick liquid film gathers to the rear side of the pillar during interfacial shearing movement, and no obvious pillar deformation appearing indicates almost zero friction force acting between pillar and substrate (Fig. 3C and movie S4).

As the NFPF pillar height changes from 10 to 30 μm, *x*_R_ increases from 13.9 to 20.5 μm, and ∆*d* increases from 3.4 to 8.0 μm accompanied with the pillar surface stretched more intensely under interfacial boundary friction (Fig. 3, E and F, and fig. S12D). This attributes to the decrease in pillar shear modulus as its height increasing. During sliding, the pillar’s deformation and separation both greatly affect the interfacial liquid film area. On BP, the liquid film area decreases rapidly to ~64% before utter separation (Fig. 3G). While on NFPF, the liquid film area increases slowly at the beginning due to the pillar being stretched and then gradually decreases. Before pillar’s utter separation, higher NFPF maintains larger liquid film area. The NFPF-30 could maintain ~95% liquid film area compared to NFPF-10 with ~88% and even ~1.5 times higher than BP-10, which indicates that a more stable interfacial liquid film with larger capillarity is maintained during sliding on NFPF-30 (Fig. 3H). Such correlated dynamic interaction created by structural deformation and interfacial liquid film distribution eventually leads to the robust liquid film and strong boundary on NFPF pillar and cricket pad.

### Interfacial stress transmission on NFPF and BP surfaces

**Fig. 4.**
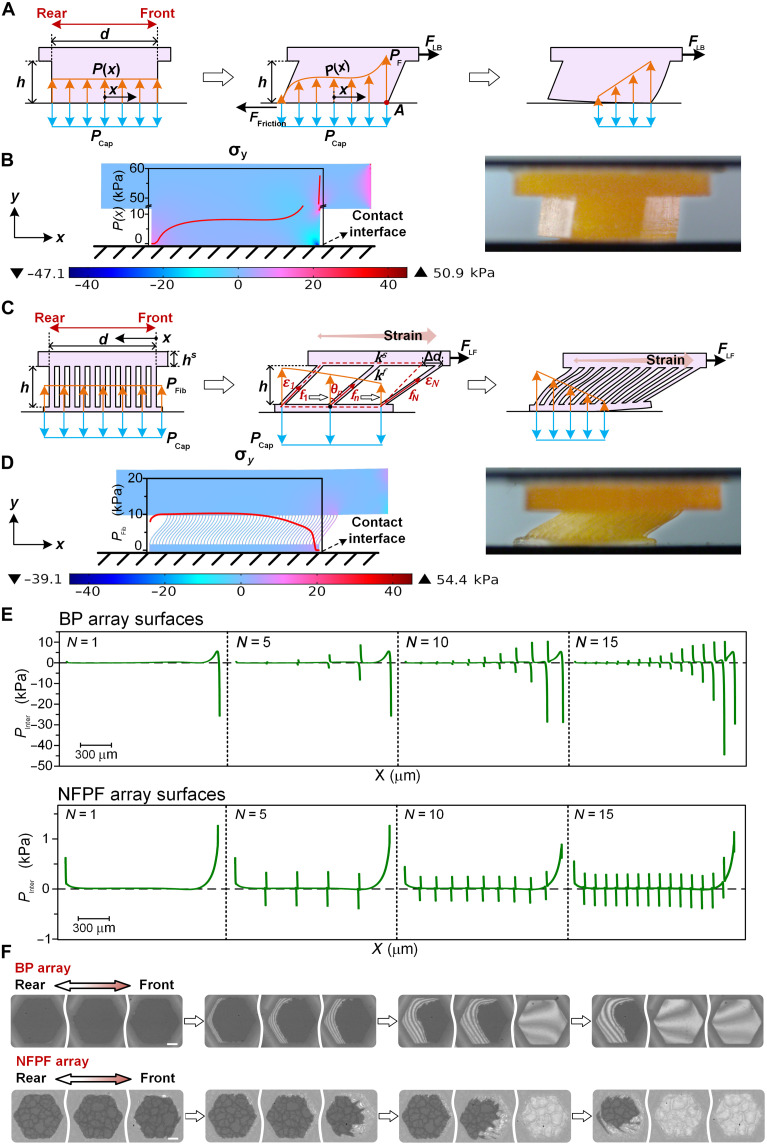
Theory and FEA simulation of stress transmission on BP and NFPF surfaces. (**A**) During sliding with lateral force *F*_LB_, the BP exhibits extrusion stress at the front side and separates from the rear side. The corner of the pillar at the front side is denoted as corner A. *h* and *d* are the pillar height and diameter, respectively. *P*_Cap_ is the capillary stress generated by nano-liquid film and *P*(*x*) is the interfacial solid-solid contact stress along the *x* axis. (**B**) Finite element analysis (FEA) simulation of the contact stress *P*(*x*) on BP and a scaled-up soft BP pillar to demonstrate the structural deformation during friction. (**C**) Schematic diagram of the stress transmission on NFPF with stretching stress appearing at the front side. From rear to front, the fibers are marked from the first to *N*th. (**D**) FEA simulation of the contact stress of NFPF and a scaled-up NFPF pillar to demonstrate the structural deformation during friction. (**E**) FEA simulation of the interfacial normal stress *P*_Inter_ of BP and NFPF array surfaces with a different number of pillars. (**F**) The process of multiple BP and NFPF separating from the substrate. Scale bars, 20 μm.

The theoretical models have been developed to analyze the interfacial stress transmission on BP and NFPF. Because of the homogeneous structure of the BP, it is not susceptible to shear deformation when the lateral force *F*_LB_ is applied. Under the bending moment formed by *F*_LB_ and pillar height *h*, the BP is deformed and inclined, which results in the front side of the pillar being extruded and the rear side of the pillar being stretched (Fig. 4, A and B). The force balance between capillary pressure *P*_Cap_ and solid-solid contact stress distribution function *P*(*x*) in the normal direction of the contact interface can be represented as

$$\int_{-\frac{d}{2}}^{\frac{d}{2}}P(x)L\;dx=\int_{-\frac{d}{2}}^{\frac{d}{2}}P_{\mathrm{Cap}}L\;dx$$ (1)

where *d* and *L* refer to the width and thickness of the pillar, respectively. Selecting corner A as the pivot point, the bending moment equilibrium formed by solid-solid contact stress *P*(*x*), external lateral force *F*_LB_, and capillary pressure *P*_Cap_ can be expressed as

$$\int_{-\frac{d}{2}}^{\frac{d}{2}}P(x)\;\left(\frac{d}{2}-x\right)L\;dx+F_{\mathrm{LB}}h=\int_{-\frac{d}{2}}^{\frac{d}{2}}P_{\mathrm{Cap}}\;\left(\frac{d}{2}-x\right)L\;dx$$ (2)

With the increase of lateral force, the contact stress *P*_F_ at the pillar front side increases, while the stress *P*_R_ at the pillar rear side decreases. When *P*_R_ drops to zero, the pillar starts to separate from the substrate along with reaching the maximum friction.

By simplifying the solid-solid contact stress distribution *P*(*x*) at the interface from *P*_R_ to *P*_F_ as a cubic function

$$P(x)=\frac{8P_{\mathrm{Cap}}}{d^{3}}\;x^{3}+P_{\mathrm{Cap}}$$ (3)

while satisfying both Eq. 1 and the boundary condition (Supplementary Text A), then the maximum lateral force *F*_LB-max_ can be represented as

$$F_{\mathrm{LB}-\max}=\frac{P_{\mathrm{Cap}}{Ld}^{2}}{10h}$$ (4)

It is noted that the maximum lateral force *F*_LB-max_ decreases with the increase of the pillar height *h*, suggesting that a high pillar height will weaken the frictional properties of the pillar surface, which is in agreement with the experimental results.

On NFPF, each thin and long fiber is more easily able to transmit tensile stress than compressive stress due to its deflection; thus, a completely different stress transmission mode appears. To establish the theoretical model, the NFPF is divided into top surface and bottom fiber array, which can be simplified as elastic springs with a linear relationship between the force and deformation (Fig. 4C). When the top surface is elongated by Δ*d* under a lateral force *F_LF_*, fibers at the pillar front side are more stretched than those at the rear side, resulting in a lower contact stress at the front side. Then, the pillar front side tends to be first separated from substrate as the lateral force increasing (Fig. 4D). Because the fibers can only transmit tensile stress, they can be considered as an elastic spring with an equivalent elasticity coefficient of *k^f^*. Define ɛ*_n_*, *f_n_*, and *θ_n_* as the axial strain, axial tension, and horizontal tilting angle of the *n*th fiber, respectively. The force balance in horizontal direction can be represented as

$$\sum_{n=1}^{N}f_{n}\;\sin\theta_{n}=F_{\mathrm{LF}}$$ (5)

where *f_n_* = *k^f^*ɛ*_n_h* and *h* is the height of the fiber (Fig. 4C and Supplementary Text B). When the pillar front side starts to detach from the substrate and slide, the surface reaches its highest friction with maximum lateral force *F*_LF-max_. For the front side fiber (the *N*th fiber), the force balance in vertical direction at the contact surface can be expressed as

$$f_{N}\cos\theta_{N}=P_{\mathrm{Cap}}*L\phi$$ (6)

where *L* is the thickness of the pillar and ϕ the diameter of the fiber. On the basis of the force equilibrium at the top surface, the maximum lateral force *F*_LF-max_ and the elongation of the top surface Δ*d* have a relation of

$$k^{s}\text{Δ}d=F_{\mathrm{LF}\text{-}\max}$$ (7)

where *k^s^* is the equivalent elasticity coefficient of the top surface. Combining Eqs. 5 to 7, the maximum lateral force *F*_LF-max_ can be derived as

$$F_{\mathrm{LF}\text{-}\max}=\frac{2P_{\mathrm{Cap}}{Lk}^{s}h\sqrt{\varepsilon_{N}(\varepsilon_{N}+2)}}{2k^{s}\alpha (1+\beta )+P_{\mathrm{Cap}}L}$$ (8)

where α = *h*/*d*, β = *w*/ϕ, and *w* refers to the spacing between fibers. As the fiber height increases, the maximum lateral force *F*_LF-max_ becomes larger, indicating that bioinspired pillars with high fiber arrays feature greater frictional properties, which is consistent with the characterization results of wet friction performance for NFPF surfaces. Nevertheless, further fiber height increasing cannot continuously enhance the boundary friction performance, which could be attributed to that the higher pillar leads to a tilting deformation during lateral sliding (fig. S9C) (*35*, *40*). A separating stress could occur at the rear side of NFPF, as in the case of BP. On the basis of the pillar height-diameter ratio of the bush cricket pad and NFPF surfaces, the best height-diameter ratio is ~0.5.

To compare the frictional performance of the NFPF and BP, ζ is defined as the ratio of the maximum lateral force on the NFPF (*F*_LF-max_) and BP (*F*_LB-max_) (Supplementary Text C), which is

$$\zeta =\frac{F_{\mathrm{LF}\text{-}\max}}{F_{\mathrm{LB}\text{-}\max}}=\frac{20k^{s}\alpha^{2}\sqrt{\varepsilon_{N}(\varepsilon_{N}+2)}}{2k^{s}\alpha (1+\beta )+P_{\mathrm{Cap}}L}$$ (9)

Numerical calculations show that the ratio ζ > 1, indicating that the NFPF surfaces exhibit better friction properties than the BP surfaces and agrees with the experimental results (table S1 and fig. S13).

Moreover, the pillar array structure could redistribute the contact stress over the entire surface to further enhance the boundary friction. Finite element analysis (FEA) simulations of surfaces with a different number of pillars were performed to analyze the contact stress distribution, where lateral displacement was applied at one side of the surface (fig. S14). On smooth surface (*N* = 1), a continuous change of interfacial normal contact stress *P*_Inter_ appears, where high extruding *P*_Inter_ forms at the front side and quickly changes into separating *P*_Inter_ along the surface (Fig. 4E, top). While on BP array surfaces, *P*_Inter_ turns into discrete distribution with each pillar being extruded at the front side and separating at the rear side, which forms a contact stress self-splitting effect. With pillar number *N* increasing, the self-splitting effect becomes more dominant and leads to increased extrusion stress on each pillar and the overall surface. However, the separation stress at each pillar’s rear side increases as well, which results in destroying of nano liquid films and failure in boundary friction (Fig. 4E, top; *N* > 1).

As for NFPF with *N* = 1, a separating *P*_Inter_ forms at the front side and decreases along the surface (Fig. 4E, bottom), which is distinctly different from the extruding pressure on BP with *N* = 1. While on NFPF array surfaces, *P*_Inter_ turns into the discrete distribution in a different way from BP array surfaces. The front pillar withstands separating *P*_Inter_ at the pillar front side, while other pillars behind are subjected to extruding *P*_Inter_ at the pillar front side and separating *P*_Inter_ at the pillar rear side. Compared with the nanofiber-embedded surface (*N* = 1), the discrete distribution of *P*_Inter_ on the NFPF array surface enhances the extrusion stress on individual pillars and the entire surface, which increases the stability of interfacial liquid films and thus achieves the robust contact at the interface.

With increased lateral force on BP, the first front pillar starts to separate from the substrate, and the adjacent pillars almost simultaneously detach from the substrate (Fig. 4F, top), which is not conducive to maintaining the solid-solid contact. The NFPF array surface will first separate from the substrate at the front pillar under continuous applied lateral force, and, after the separation of the front pillar, the subsequent pillars will then detach from the substrate one by one (Fig. 4F, bottom), which is beneficial for improving the stability of the interfacial contact.

### Application of bioinspired NFPF surfaces

**Fig. 5.**
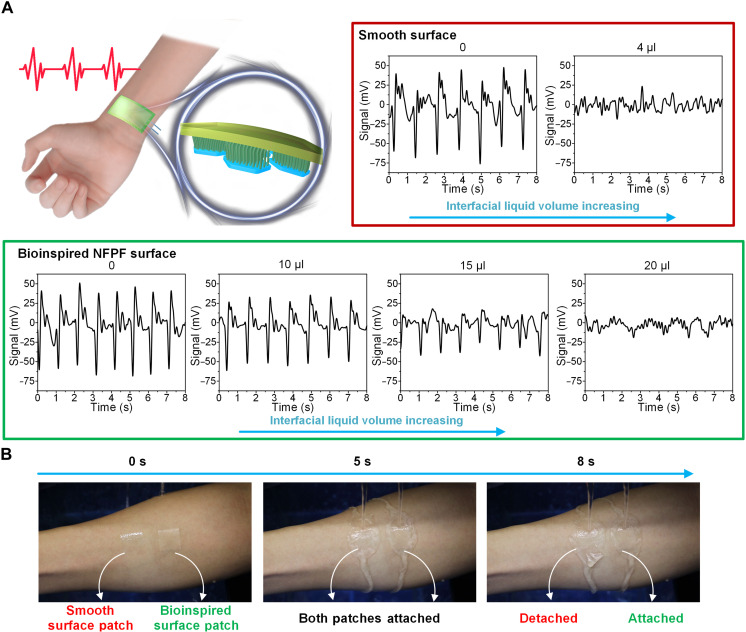
Adhesion performance of bioinspired surface in the application of bio-signal monitoring. (**A**) Schematic illustration of detecting pulse signals with the bioinspired NFPF skin patch combined with a piezoelectric sensor. Pulse signals can be distinguished with a wider range of interfacial liquid volume on the bioinspired surface than that of a smooth surface patch, which shows that the bioinspired surface has stronger water tolerance for signal detecting. (**B**) Testing flow tolerance of bioinspired and smooth surface patches in flowing water (*Q* ≈ 90 ml cm^−2^ s^−1^) on a wet forearm shows that the bioinspired surface patch can steadily adhere to wet human skin.

For potential applications to wearable flexible electronics for medical and health monitoring, the bioinspired NFPF skin patch was integrated with a commercial piezoelectric sensor for detecting pulse signals (fig. S15). The bioinspired NFPF patch exhibits better water tolerance than the smooth patch (Fig. 5A), which can avoid degradation of detection accuracy or detection failure to some extent due to the existence of liquid on biological tissues or organs. To demonstrate the wet attachment property of the NFPF patch, the flowing water resistance tests of the bioinspired patch were conducted on the volunteer’s forearm. Compared to the smooth patch, the NFPF patch can resist detachment from the volunteer’s arm against flowing water (*Q* ≈ 90 ml cm^−2^ s^−1^) for a longer period (Fig. 5B and movie S5), demonstrating the usability of bioinspired patches in more complex wet environments. This bioinspired NFPF patch can also be easily peeled off from the skin even without any pain to the skin generally caused by vacuum suckers or tissue adhesives (fig. S16).

## DISCUSSION

A strong wet friction surface has been discovered on the Chinese bush cricket pad with its unique hierarchical micro-nano fibrous pillar structure. Under such inspiration, NFPF has been built for wearable electronics. Unique interfacial stress shifting effect was found in situ observation of interfacial contact dynamic behaviors as compared with BP in that both initial separating region of NFPF was changed from the rear side of the pillar to the front side and its deformation mode also varied from compressing to stretching during sliding. Such interfacial contact stress shifting effect largely decreases the separation stress exerted on interfacial liquid film and increases the liquid film area by about 50% to greatly enhance the stability of nano-liquid bridges. Self-splitting effect of interfacial liquid and contact stress of NFPF array surface could further guard such interfacial contact stress shifting effect to ensure ~1.9 times enhancement in stronger wet boundary friction. Last, the theory of contact stress shifting and wet friction enhancing has been established, and the applications on wearable electronics have been validated.

## MATERIALS AND METHODS

### Sample preparation and structural characteristics of Chinese bush cricket attachment pads

Chinese bush crickets (*G. gratiosa*, *Tettigoniidae*) were purchased from Mingyage company (Shandong province, China). The tarsus of the cricket was separated and cleaned with 0.1 M phosphate-buffered solution and then immersed in 2.5% glutaraldehyde solution at pH 7.4 for 5 days. After that, the sample was dehydrated in graded ethanol and lastly dried with a critical point dryer (Tousimis 931 Series, USA). To prepare the frozen-fractured biological sample, the fresh tarsus was glued to a copper holder and then frozen rapidly in liquid nitrogen and cryo-transferred into the stage of the freezing preparation system (PP3000T, QUORUM). The sample was fractured by a cold knife and sublimated at −90°C for 10 min, then cryo-coated with Pt-sputter (10 mA, 2 min), and cryo-transferred into the cryo-scanning electron microscope using the cryo-transmission rod. A focused ion beam scanning electron microscope (Helios G4 CX, Czech Republic) and a dual-beam scanning electron microscope (Helios NanoLab 600i, USA) were applied to observe the micro-nano structures of the critical point dried sample and the cross-sectional morphology of the frozen sample, respectively. All the animal protocols were approved by the Biological and Medical Ethics Committee of Beihang University with the authorization number BM20210089.

### Liquid film characteristics and friction tests for Chinese bush cricket attachment pads

A living cricket was inverted, then the back of the insect body was taped to the platform, and the tarsus was fixed to the stage with paraffin (all tarsi of the insect consist of four tarsomeres, and friction tests were conducted on the second tarsomere near the end of the tarsus). The glass substrate was mounted on a motorized XYZ-translation stage integrated with a two-dimensional force sensor and parallel to the sample stage, ensuring that the glass substrate was only in contact with the attachment pads. Then, the glass substrate was pushed toward the pads to provide an appropriate normal force of about 1 mN. During the friction test, the glass substrate was moved horizontally toward the distal direction of pads with a sliding speed of 200 μm s^−1^, as shown in fig. S3A. After one test was completed, the glass substrate was lifted vertically and moved to a clean area to repeat the above test process. The friction of the attachment pads was recorded with a force sensor (LH-72-50, Liheng sensors Co. Ltd., China). The mucus film state at the contact interface was captured using a microscope (BX51, Olympus) attached to a high-speed camera (Mini UX100, Photron, Japan).

### Fabrication of bioinspired surfaces

The bioinspired BP surfaces (1 cm by 1 cm) were obtained by replicating photoresist AZ4620 (AZ Electronic Materials) models with polydimethylsiloxane (PDMS; Sylgard 184, Dow Corning) (fig. S4B). The PSAN surfaces (1 cm by 1 cm) with pillars composed of self-assembled nanofibers were fabricated by replicating the parylene-coated AAO template (Shenzhen Topmembranes) (fig. S4C). The bioinspired NFPF surfaces (1 cm by 1 cm) with hierarchical nanofibers were fabricated by pouring PDMS onto parylene-coated AAO templates and curing at 100°C for 2 hours and then submerged sequentially in 5% NaOH aqueous solution and 20% CuCl_2_ aqueous solution to remove the backside oxide layer and aluminum base. After washing the sample in deionized water, the sample was immersed in 40°C 3% phosphoric acid solution for a setting period to partially corrode the AAO layer. Depending on the thickness of AAO, 70, 100, and, 110 min corroding time was chosen for 10-, 20-, and 30-μm thickness AAO, respectively. Then, a thin PDMS layer was spin-coated onto the top of the sample and cured at 100°C for 2 hours, following with photolithography to prepare a hexagonal shaped photoresist (AZ4620) for the reactive ion etching process on the exposed PDMS layer. Last, the sample was immersed in ethanol and a 5% NaOH aqueous solution to remove the photoresist AZ4620 and residual AAO, respectively (fig. S4A).

### Liquid film characteristics and friction tests for bioinspired surfaces

Liquid film observations for bioinspired surfaces were performed with the same equipment for cricket attachment pads characterization. Five times of tests were repeatedly performed at ambient temperature. Before each test, the surfaces were modified to hydrophilic using oxygen plasma. A volume of 1.5 μl of deionized water was injected onto bioinspired surfaces. Then, the glass substrate was put on the bioinspired surface without applying additional vertical force. The glass substrate was moved horizontally to conduct the friction test. After completing a test, the glass substrate was lifted vertically and moved to a clean area to repeat the above test process until the water on the contact area disappeared. The shear stress was obtained by dividing the friction force by the bioinspired surface area of 1 cm by 1 cm. The pillars’ lateral deformation during friction tests was captured by a microscope (fig. S3B). Dark areas denote the liquid between the pillar and the substrate. The lateral deformation of pillars indicated that they were subjected to friction from the substrate. According to the liquid distribution and the deformation of pillars, the friction state of the pillars was classified into wet, hybrid, and boundary states during friction tests.

## Supplementary Materials

### This PDF file includes:

Supplementary Texts A to C Figs. S1 to S16 Table S1

## References

[1] D.-H. Kim, J.-H. Ahn, W. M. Choi, H.-S. Kim, T.-H. Kim, J. Song, Y. Y. Huang, Z. Liu, C. Lu, J. A. Rogers, Stretchable and foldable silicon integrated circuits. Science 320, 507–511 (2008). 18369106 10.1126/science.1154367

[2] K.-I. Jang, H. N. Jung, J. W. Lee, S. Xu, Y. H. Liu, Y. Ma, J.-W. Jeong, Y. M. Song, J. Kim, B. H. Kim, A. Banks, J. W. Kwak, Y. Yang, D. Shi, Z. Wei, X. Feng, U. Paik, Y. Huang, R. Ghaffari, J. A. Rogers, Ferromagnetic, folded electrode composite as a soft interface to the skin for long-term electrophysiological recording. Adv. Funct. Mater. 26, 7281–7290 (2016). 28413376 10.1002/adfm.201603146 PMC5390688

[3] J. Choi, D. Kang, S. Han, S. B. Kim, J. A. Rogers, Thin, soft, skin-mounted microfluidic networks with capillary bursting valves for chrono-sampling of sweat. Adv. Healthc. Mater. 6, 1601355 (2017). 10.1002/adhm.201601355 28105745

[4] S. Emaminejad, W. Gao, E. Wu, Z. A. Davies, H. Y. Y. Nyein, S. Challa, S. P. Ryan, H. M. Fahad, K. Chen, Z. Shahpar, S. Talebi, C. Milla, A. Javey, R. W. Davis, Autonomous sweat extraction and analysis applied to cystic fibrosis and glucose monitoring using a fully integrated wearable platform. Proc. Natl. Acad. Sci. U.S.A. 114, 4625–4630 (2017). 28416667 10.1073/pnas.1701740114 PMC5422810

[5] J. Choi, R. Ghaffari, L. B. Baker, J. A. Rogers, Skin-interfaced systems for sweat collection and analytics. Sci. Adv. 4, eaar3921 (2018). 29487915 10.1126/sciadv.aar3921 PMC5817925

[6] M. Pal, A. Giri, D. W. Kim, S. Shin, M. Kong, K. Thiyagarajan, J. Kwak, O. F. N. Okello, S.-Y. Choi, U. Jeong, Fabrication of foldable metal interconnections by hybridizing with amorphous carbon ultrathin anisotropic conductive film. ACS Nano 13, 7175–7184 (2019). 31149801 10.1021/acsnano.9b02649

[7] J. H. Peters, G. D. Gibbons, J. T. Innes, K. E. Nichols, M. E. Front, S. R. Roby, E. C. Ellison, Complications of laparoscopic cholecystectomy. Surgery 110, 769–777 (1991). 1833848

[8] N. J. Soper, D. L. Dunnegan, Does intraoperative gallbladder perforation influence the early outcome of laparoscopic cholecystectomy? Surg. Laparosc. Endosc. 1, 156–161 (1991). 1669395

[9] S. Johnston, K. O'Malley, G. McEntee, P. Grace, E. Smyth, D. Bouchier-Hayes, The need to retrieve the dropped stone during laparoscopic cholecystectomy. Am. J. Surg. 167, 608–610 (1994). 8209938 10.1016/0002-9610(94)90108-2

[10] P. Schrenk, R. Woisetschlager, R. Rieger, W. Wayand, Mechanism, management, and prevention of laparoscopic bowel injuries. Gastrointest. Endosc. 43, 572–574 (1996). 8781935 10.1016/s0016-5107(96)70193-1

[11] J. T. Bishoff, M. E. Allaf, W. Kirkels, R. G. Moore, L. R. Kavoussi, F. Schroder, Laparoscopic bowel injury: Incidence and clinical presentation. J. Urol. 161, 887–890 (1999). 10022706 10.1016/s0022-5347(01)61797-x

[12] L. Zhang, S. Zhao, X. Zhou, X. Jing, Y. Zhou, Y. Wang, Y. Zhu, X. Liu, Z. Zhao, D. Zhang, L. Feng, H. Chen, A magnetic-driven multi-motion robot with position/orientation sensing capability. Research 6, 0177 (2023). 10.34133/research.0177 PMC11778601 39882544

[13] H. Lee, S. M. Dellatore, W. M. Miller, P. B. Messersmith, Mussel-inspired surface chemistry for multifunctional coatings. Science 318, 426–430 (2007). 17947576 10.1126/science.1147241 PMC2601629

[14] H. Lee, B. P. Lee, P. B. Messersmith, A reversible wet/dry adhesive inspired by mussels and geckos. Nature 448, 338–341 (2007). 17637666 10.1038/nature05968

[15] J. Li, A. D. Celiz, J. Yang, Q. Yang, I. Wamala, W. Whyte, B. R. Seo, N. V. Vasilyev, J. J. Vlassak, Z. Suo, D. J. Mooney, Tough adhesives for diverse wet surfaces. Science 357, 378–381 (2017). 28751604 10.1126/science.aah6362 PMC5905340

[16] J. Wunderer, B. Lengerer, R. Pjeta, P. Bertemes, L. Kremser, H. Lindner, T. Ederth, M. W. Hess, D. Stock, W. Salvenmoser, P. Ladurner, A mechanism for temporary bioadhesion. Proc. Natl. Acad. Sci. U.S.A. 116, 4297–4306 (2019). 30782790 10.1073/pnas.1814230116 PMC6410801

[17] Q. Zhao, D. W. Lee, B. K. Ahn, S. Seo, Y. Kaufman, J. N. Israelachvili, J. H. Waite, Underwater contact adhesion and microarchitecture in polyelectrolyte complexes actuated by solvent exchange. Nat. Mater. 15, 407–412 (2016). 26779881 10.1038/nmat4539 PMC4939084

[18] S. Baik, D. W. Kim, Y. Park, T.-J. Lee, S. Ho Bhang, C. Pang, A wet-tolerant adhesive patch inspired by protuberances in suction cups of octopi. Nature 546, 396–400 (2017). 28617467 10.1038/nature22382

[19] D. W. Kim, S. Baik, H. Min, S. Chun, H. J. Lee, K. H. Kim, J. Y. Lee, C. Pang, Highly permeable skin patch with conductive hierarchical architectures inspired by amphibians and octopi for omnidirectionally enhanced wet adhesion. Adv. Funct. Mater. 29, 1807614 (2019).

[20] S. Song, D.-M. Drotlef, C. Majidi, M. Sitti, Controllable load sharing for soft adhesive interfaces on three-dimensional surfaces. Proc. Natl. Acad. Sci. U.S.A. 114, E4344–E4353 (2017). 28507143 10.1073/pnas.1620344114 PMC5465890

[21] S. Baik, J. Kim, H. J. Lee, T. H. Lee, C. Pang, Highly adaptable and biocompatible octopus-like adhesive patches with meniscus-controlled unfoldable 3D microtips for underwater surface and hairy skin. Adv. Sci. 5, 1800100 (2018). 10.1002/advs.201800100 PMC6097001 30128235

[22] Y. Wu, X. Li, H. Tian, D. Wang, J. Zhang, L. Wang, J. Shao, Microtemplated electrowetting for fabrication of shape-controllable microdomes in extruded microsucker arrays toward octopus-inspired dry/wet adhesion. Adv. Funct. Mater. 33, 2210562 (2023).

[23] H. Yuk, C. E. Varela, C. S. Nabzdyk, X. Mao, R. F. Padera, E. T. Roche, X. Zhao, Dry double-sided tape for adhesion of wet tissues and devices. Nature 575, 169–174 (2019). 31666696 10.1038/s41586-019-1710-5

[24] X. Chen, H. Yuk, J. Wu, C. S. Nabzdyk, X. Zhao, Instant tough bioadhesive with triggerable benign detachment. Proc. Natl. Acad. Sci. U.S.A. 117, 15497–15503 (2020). 32576692 10.1073/pnas.2006389117 PMC7376570

[25] J. Deng, H. Yuk, J. Wu, C. E. Varela, X. Chen, E. T. Roche, C. F. Guo, X. Zhao, Electrical bioadhesive interface for bioelectronics. Nat. Mater. 20, 229–236 (2021). 32989277 10.1038/s41563-020-00814-2

[26] J. Yang, R. Bai, B. Chen, Z. Suo, Hydrogel adhesion: A supramolecular synergy of chemistry, topology, and mechanics. Adv. Funct. Mater. 30, 1901693 (2020).

[27] X. Shi, P. Wu, A smart patch with on-demand detachable adhesion for bioelectronics. Small 17, 2101220 (2021). 10.1002/smll.202101220 34105250

[28] Y. Xue, J. Zhang, X. Chen, J. Zhang, G. Chen, K. Zhang, J. Lin, C. Guo, J. Liu, Trigger-detachable hydrogel adhesives for bioelectronic interfaces. Adv. Funct. Mater. 31, 2106446 (2021).

[29] G. Tian, D. Yang, C. Liang, Y. Liu, J. Chen, Q. Zhao, S. Tang, J. Huang, P. Xu, Z. Liu, D. Qi, A nonswelling hydrogel with regenerable high wet tissue adhesion for bioelectronics. Adv. Mater. 35, e2212302 (2023). 36739173 10.1002/adma.202212302

[30] S. Gorb, M. Scherge, Biological microtribology: Anisotropy in frictional forces of orthopteran attachment pads reflects the ultrastructure of a highly deformable material. Proc. Biol. Sci. 267, 1239–1244 (2000). 10902690 10.1098/rspb.2000.1133 PMC1690659

[31] W. Federle, W. J. P. Barnes, W. Baumgartner, P. Drechsler, J. M. Smith, Wet but not slippery: Boundary friction in tree frog adhesive toe pads. J. R. Soc. Interface 3, 689–697 (2006). 16971337 10.1098/rsif.2006.0135 PMC1664653

[32] H. Chen, L. Zhang, D. Zhang, P. Zhang, Z. Han, Bioinspired surface for surgical graspers based on the strong wet friction of tree frog toe pads. ACS Appl. Mater. Interfaces 7, 13987–13995 (2015). 26053597 10.1021/acsami.5b03039

[33] Y. Wang, L. Zhang, Y. Guo, Y. Gan, G. Liu, D. Zhang, H. Chen, Air bubble bridge-based bioinspired underwater adhesion. Small 17, e2103423 (2021). 34554641 10.1002/smll.202103423

[34] L. Zhang, H. Chen, Y. Guo, Y. Wang, Y. Jiang, D. Zhang, L. Ma, J. Luo, L. Jiang, Micro–nano hierarchical structure enhanced strong wet friction surface inspired by tree frogs. Adv. Sci. 7, 2001125 (2020). 10.1002/advs.202001125 PMC7578903 33101853

[35] L. Zhang, Y. Guo, Y. Wang, J. Liang, Y. Zhou, X. Liu, D. Zhang, H. Chen, Multi-dimensional self-splitting behaviors for improving wet attachment on nonuniform bioinspired pillar surface. Adv. Funct. Mater. 32, 2205804 (2022).

[36] L. Zhang, Y. Wang, Z. Wang, G. Liu, Y. Guo, X. Liu, D. Zhang, L. Jiang, H. Chen, Liquid/air dynamic behaviors and regulation mechanisms for bioinspired surface. Appl. Phys. Rev. 9, 041315 (2022).

[37] N. J. Glassmaker, A. Jagota, C. Y. Hui, W. L. Noderer, M. K. Chaudhury, Biologically inspired crack trapping for enhanced adhesion. Proc. Natl. Acad. Sci. U.S.A. 104, 10786–10791 (2007). 17581870 10.1073/pnas.0703762104 PMC1904130

[38] L. Xue, B. Sanz, A. Luo, K. T. Turner, X. Wang, D. Tan, R. Zhang, H. Du, M. Steinhart, C. Mijangos, M. Guttmann, M. Kappl, A. del Campo, Hybrid surface patterns mimicking the design of the adhesive toe pad of tree frog. ACS Nano 11, 9711–9719 (2017). 28885831 10.1021/acsnano.7b04994 PMC5656980

[39] H. Tian, D. Wang, Y. Zhang, Y. Jiang, T. Liu, X. Li, C. Wang, X. Chen, J. Shao, Core-shell dry adhesives for rough surfaces via electrically responsive self-growing strategy. Nat. Commun. 13, 7659 (2022). 36496484 10.1038/s41467-022-35436-6 PMC9741600

[40] B. Murarash, Y. Itovich, M. Varenberg, Tuning elastomer friction by hexagonal surface patterning. Soft Matter 7, 5553–5557 (2011).

