# Supplementary material for: Nanofiber embedded bioinspired strong wet friction surface
Source: Sci Adv. 2023 Oct 12;9(41):eadi4843. doi: 10.1126/sciadv.adi4843 (PMC10569708; doi:10.1126/sciadv.adi4843)
Supplement: Supplementary file 1 — Supplementary Texts A to C Figs. S1 to S16 Table S1 [file sciadv.adi4843_sm.pdf]

Science Advances
AAAS

Supplementary Materials for

Nanofiber embedded bioinspired strong wet friction surface

Yurun Guo *et al.*

Corresponding author: Huawei Chen, chenhw75@buaa.edu.cn; Liwen Zhang, lwzhang@buaa.edu.cn

Sci. Adv. **9**, eadi4843 (2023)
DOI: 10.1126/sciadv.adi4843

The PDF file includes:

Supplementary Texts A to C
Figs. S1 to S16
Table S1

Other Supplementary Material for this manuscript includes the following:

Movies S1 to S5

Supplementary Text

A. On BP, the relationship between the solid contact stress, capillary pressure, and external lateral force

Due to the homogeneous structure of the BP, the BP is deformed and inclined under the bending moment formed by the lateral force F_{LB} and pillar height h (Fig. 4A). The force balance between capillary pressure P_{Cap} and solid-solid contact stress distribution function $P(x)$ in the normal direction of the contact interface can be represented as

$$\int_{-\frac{d}{2}}^{\frac{d}{2}} P(x)L\,\mathrm{d}x = \int_{-\frac{d}{2}}^{\frac{d}{2}} P_{Cap}L\,\mathrm{d}x \qquad \text{S1}$$

where d and L refer to the width and thickness of the pillar, respectively. Selecting corner A as the pivot point, the bending moment equilibrium formed by solid-solid contact stress $P(x)$, external lateral force F_{LB}, and capillary pressure P_{Cap} can be expressed as

$$\int_{-\frac{d}{2}}^{\frac{d}{2}} P(x)\left(\frac{d}{2}-x\right)L\,\mathrm{d}x + F_{LB}h = \int_{-\frac{d}{2}}^{\frac{d}{2}} P_{Cap}\left(\frac{d}{2}-x\right)L\,\mathrm{d}x \qquad \text{S2}$$

With the increase of lateral force, the contact stress P_F at pillar front side increases, while the stress P_R at pillar rear side decreases. When P_R drops to zero, the pillar starts to separate from the substrate along with reaching the maximum friction. $P(x)$ at the interface from P_R to P_F can be simplified as a cubic function satisfying both equation S1 and the boundary condition $P(x = -d/2) = 0$. Combining equation S1 and the boundary condition, $P(x)$ can be represented as

$$P(x) = \frac{8P_{Cap}}{d^3}x^3 + P_{Cap} \qquad \text{S3}$$

At this point, the lateral force reaches the maximum F_{LB-max}. Substituting equation S3 into equation S2, yields

$$\int_{-\frac{d}{2}}^{\frac{d}{2}} \left(\frac{8P_{Cap}}{d^3}x^3 + P_{Cap}\right)\left(\frac{d}{2}-x\right)L\,\mathrm{d}x + F_{LB-max}h = \int_{-\frac{d}{2}}^{\frac{d}{2}} P_{Cap}\left(\frac{d}{2}-x\right)L\,\mathrm{d}x$$

$$L\left(\frac{P_{Cap}}{d^2}x^4 + \frac{P_{Cap}d}{2}x - \frac{8P_{Cap}}{5d^3}x^5 - \frac{P_{Cap}}{2}x^2\right)\Bigg|_{-\frac{d}{2}}^{\frac{d}{2}} + F_{LB-max}h = P_{Cap}L\left(\frac{d}{2}x - \frac{1}{2}x^2\right)\Bigg|_{-\frac{d}{2}}^{\frac{d}{2}}$$

$$\frac{2}{5}P_{cap}Ld^2 + F_{LB-max}h = \frac{P_{cap}}{2}Ld^2$$

Then the maximum lateral force F_{LB-max} can be derived as

$$F_{LB-max} = \frac{P_{Cap}Ld^2}{10h} \qquad \text{S4}$$

It is noted that the maximum lateral force F_{LB-max} decreases with the increase of the pillar height h, suggesting that a high pillar height will weaken the frictional properties of the pillar surface, which is in agreement with the experimental results.

B. On NFPF, the relationship between the solid contact stress, capillary pressure, and external lateral force

To establish the theoretical model of NFPF stress transmission, the bioinspired pillar surface is divided into the upper surface and lower fiber array (Fig. 4C), which have a linear relationship between the force and deformation, similar to elastic springs.

Based on the linear relationship between the axial tensile deformation and the axial tension of the fiber, the fiber can be considered as an elastic spring with an equivalent elasticity coefficient of k^f. Define ε_n, f_n as the axial strain and axial tension of the nth fiber, respectively. The axial tension of the nth fiber f_n can be presented as

$$f_n = k^f \varepsilon_n h$$

where $\varepsilon_n = \Delta h/h$ in which Δh denotes the nth fiber axial elongation.

Define θ_n as the horizontal tilting angle of the nth fiber. According to the geometric relationship shown in the diagram (Fig. 4C), $sin\ \theta_n$ and $cos\ \theta_n$ can be represented as

$$\sin\theta_n = \frac{\sqrt{\varepsilon_n(\varepsilon_n + 2)}}{\varepsilon_n + 1}$$

$$\cos\theta_n = \frac{1}{\varepsilon_n + 1}$$

The force balance at the contact interface in horizontal direction (Fig.4C) can be expressed as

$$\sum_{n=1}^{N} f_n sin\ \theta_n = F_{friction} = F_{LF} \qquad \text{S5}$$

By simplifying the strain variation from the 1st to the Nth fiber as a linear change, then equation S5 can be expressed as

$$\frac{N}{2}\left(k^f \varepsilon_1 h \frac{\sqrt{\varepsilon_1(\varepsilon_1 + 2)}}{\varepsilon_1 + 1} + k^f \varepsilon_N h \frac{\sqrt{\varepsilon_N(\varepsilon_N + 2)}}{\varepsilon_N + 1}\right) = F_{LF} \qquad \text{S6}$$

where $N = d/(\phi + w)$, in which ϕ is the diameter of the fiber, and w refers to the spacing between fibers.

When the pillar front end starts to detach from the substrate and slide, the pillar surface reaches its highest friction with maximum lateral force $F_{LF\text{-}max}$. At this point, the normal force balance at the contact interface for the most anterior fiber, i.e., the Nth fiber, can be represented as $f_N\ cos\ \theta_N = P_{Cap} * L\phi$, i.e.,

$$k^f \varepsilon_N h \frac{1}{\varepsilon_N + 1} = P_{Cap} * L\phi \qquad \text{S7}$$

where P_{cap} is the capillary pressure and L is the thickness of the pillar.

Based on the force equilibrium at the upper surface, the maximum lateral force $F_{LF\text{-}max}$ and the elongation Δd of the upper surface have a relation of

$$k^s \Delta d = F_{LF-max} \qquad \text{S8}$$

where k^s represents the equivalent elasticity coefficient of the upper surface. According to the geometric relationship shown in the diagram (Fig. 4C), Δd can be represented as

$$\Delta d = h\sqrt{\varepsilon_N(\varepsilon_N + 2)} - h\sqrt{\varepsilon_1(\varepsilon_1 + 2)}$$

where ε_N and ε_1 denote the strain of the Nth and 1st fiber, respectively. Then equation S8 can be rewritten as

$$k^s\left(h\sqrt{\varepsilon_N(\varepsilon_N + 2)} - h\sqrt{\varepsilon_1(\varepsilon_1 + 2)}\right) = F_{LF-max} \qquad \text{S9}$$

Combining equations S6, S7 and S9, the maximum lateral force $F_{LF\text{-}max}$ can be derived as

$$F_{LF-max} = \frac{2P_{Cap}Lk^s h\sqrt{\varepsilon_N(\varepsilon_N + 2)}}{2k^s\alpha(1 + \beta) + P_{Cap}L} \qquad \text{S10}$$

where $\alpha = h/d$, $\beta = w/\phi$. As the fiber height increases, the maximum lateral force $F_{LF\text{-}max}$ becomes larger, indicating that bioinspired pillars with high fiber arrays feature greater frictional properties.

C. Comparison of the theoretical maximum friction between the NFPF surface and the BP surface

To compare the theoretical frictional performance of the NFPF and BP, ζ is defined as the ratio between the maximum friction on the NFPF and BP, i.e., the ratio of the maximum lateral force on the NFPF ($F_{LF\text{-}max}$) to that on the BP ($F_{LB\text{-}max}$). Combining equations S4 and S10, leads to

$$\zeta = \frac{F_{LF-max}}{F_{LB-max}} = \frac{20k^s\alpha^2\sqrt{\varepsilon_N(\varepsilon_N+2)}}{2k^s\alpha(1+\beta)+P_{Cap}L} \qquad \text{S11}$$

Calculation of k^s

Define h^s as the height of the upper surface, according to the relationship $\sigma = E\varepsilon$ of the upper surface, i.e., $\frac{k^f\Delta d}{Lh^s} = E\frac{\Delta d}{d}$, then k^s can be represented as

$$k^s = E\frac{Lh^s}{d}$$

where E represents the elastic modulus of the upper surface.

Calculation of ε_N

According to the stress-strain relationship of the fiber $\sigma = E\varepsilon$, i.e., $\frac{k^f\Delta h}{L\phi} = E\frac{\Delta h}{h}$, then k^f can be represented as

$$k^f = E\frac{L\phi}{h}$$

Substituting the above equation into equation S7, the ε_N can be derived as

$$\varepsilon_N = \frac{P_{Cap}}{E - P_{Cap}}$$

where E represents the elastic modulus of the fiber.

For numerical calculations, the elastic modulus E of PDMS is taken to be 1 MPa, and P_{cap} is assumed to be the capillary pressure of a 200 nm thick liquid film, about 700 kPa. The structural parameters of bioinspired surfaces are listed as below Table S1.

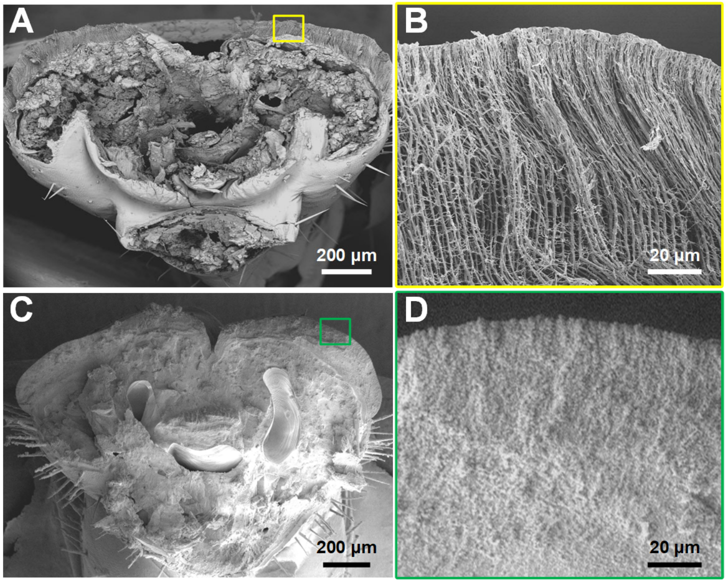

Fig. S1. Internal structural characteristics of the Chinese bush cricket attachment pads. (**A, B**) SEM for the Chinese bush cricket attachment pad after critical point drying. (**C, D**) Cryo-SEM for frozen fractured biological sample.

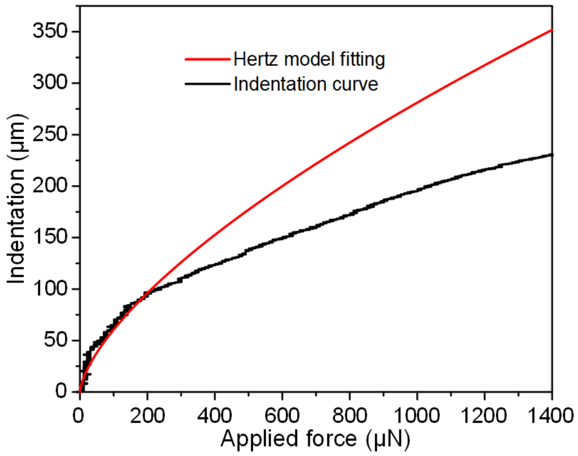

Fig. S2. Characterization of the effective elastic modulus of the Chinese bush cricket attachment pad. The indentation of the pad versus applied force. The red line is the indentation data fitted according to the Hertz model theory. The indentation of the pad was calculated by subtracting the displacement of the hard surface from the displacement of the pad at the same spring deflection to obtain the indentation of the pad under the force corresponding to that deflection. The Hertz theory predicts the indentation on the pad under an external force F_n to be $\delta = K^{-\frac{2}{3}} R^{-\frac{1}{3}} F_n^{\frac{2}{3}}$, where R is the average radius of curvature of the pad. The effective elastic modulus K of ~15 kPa for the pad was found by fitting the data for the indentation curve.

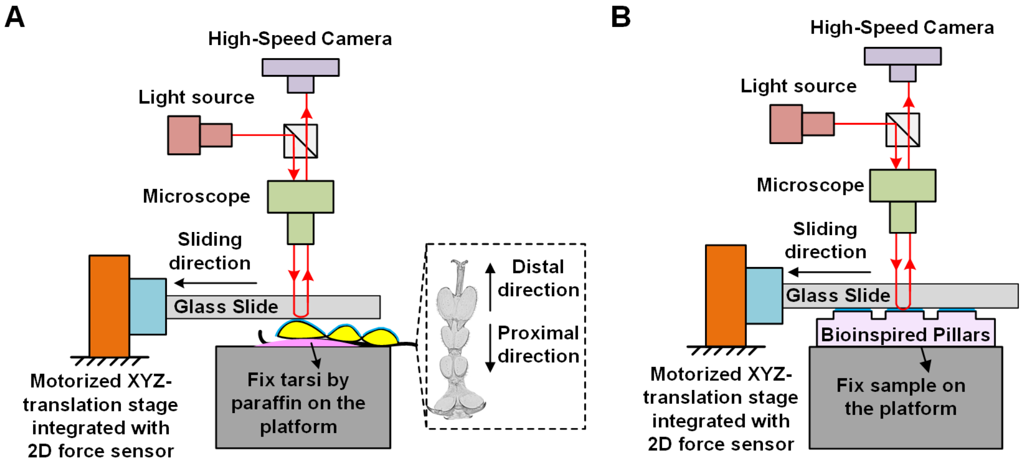

Fig. S3. *In-situ* characterization setup of interfacial liquid film movement and pillar deformation during the friction test. (**A**) Schematic illustration of performing successive friction tests for attachment pads and observing the dynamic behavior of mucus. (**B**) Schematic illustration of observing the deformation of micropillars during friction tests for bioinspired surfaces.

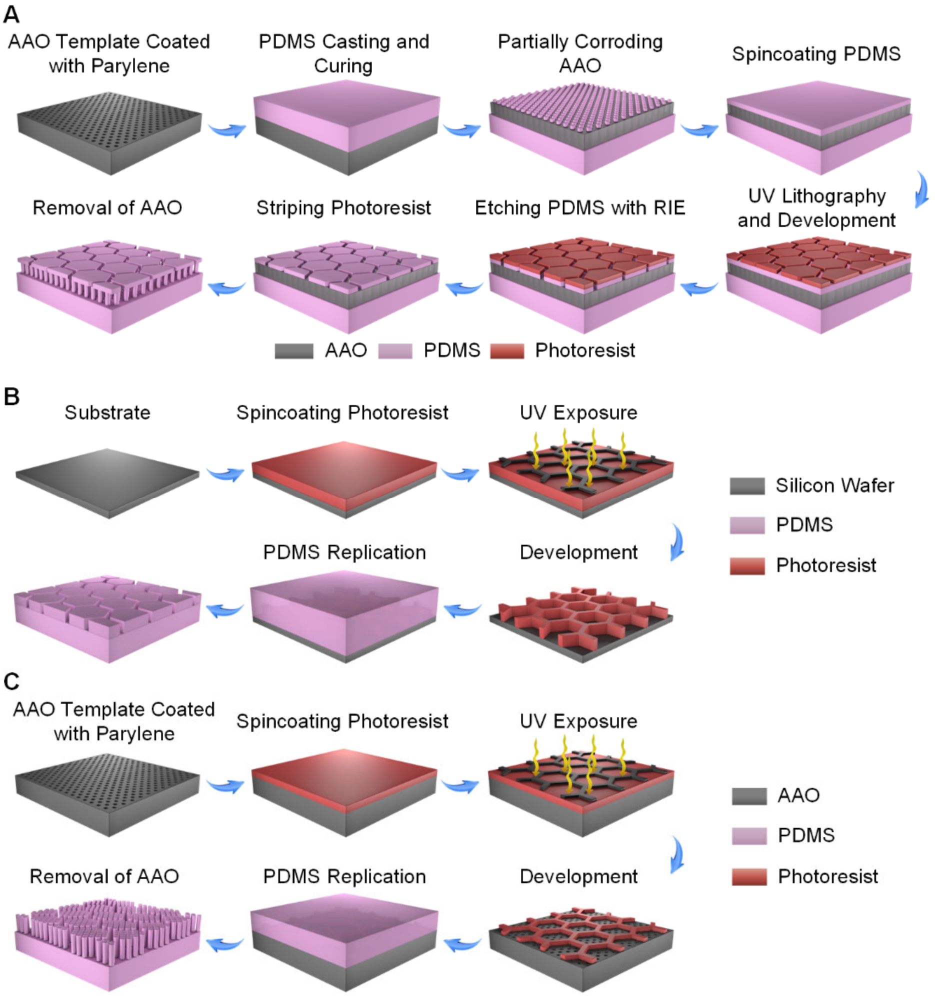

Fig. S4. Schematic illustration of the fabrication procedures of bioinspired structures. (**A, B, C**) Fabrication of NFPF, BP, and PSAN surfaces, respectively.

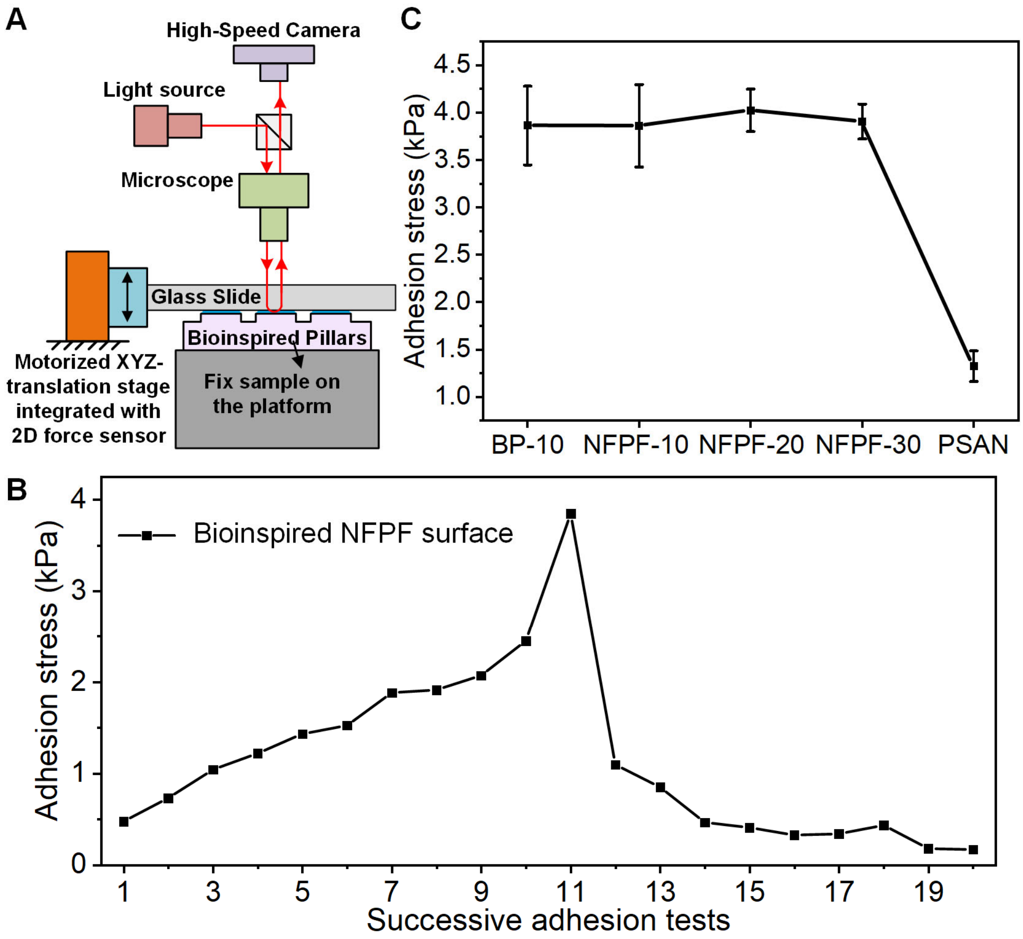

Fig. S5. *In-situ* characterization setup of normal adhesion tests for different surfaces and interfacial liquid film movement on NFPF surface during the separation process. (**A**) Schematic illustration of performing normal adhesion tests for bioinspired surfaces and observing the dynamic behavior of liquid film. (**B**) The successive normal adhesion stress of NFPF surface. (**C**) The maximum adhesion stress of different surfaces.

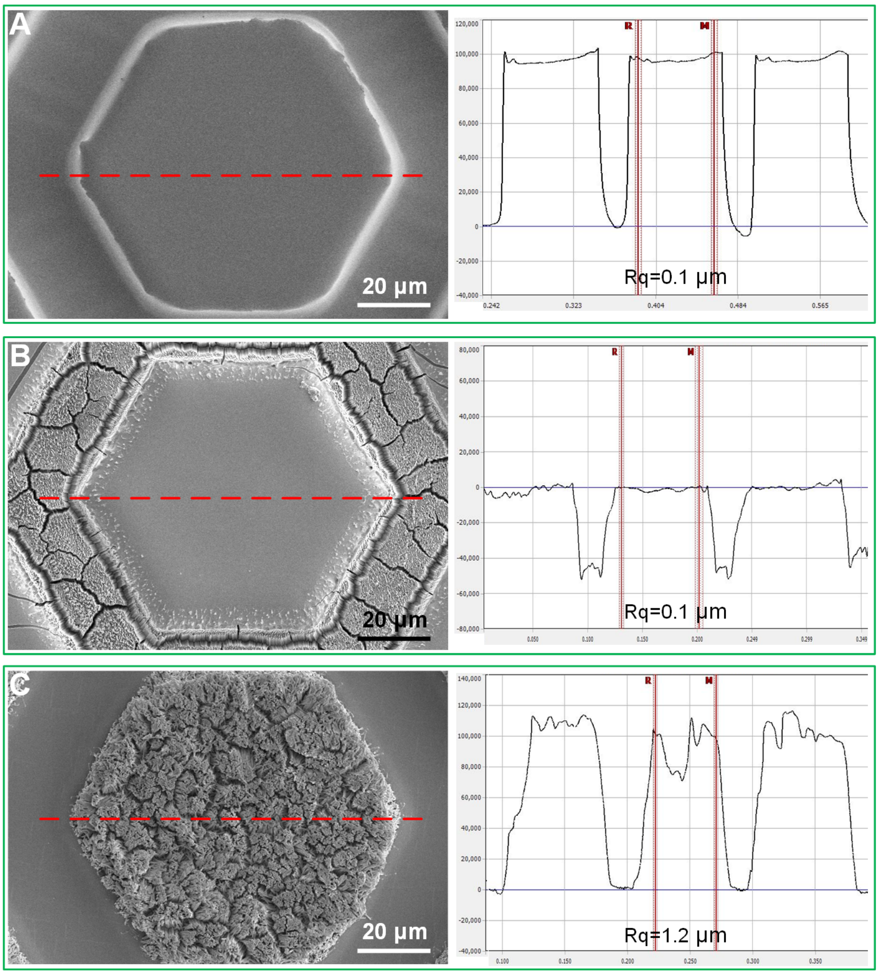

Fig. S6. Surface roughness of different pillars. (**A, B, C**) SEM images of BP, PSAN, and NFPF surfaces, and corresponding surface roughness.

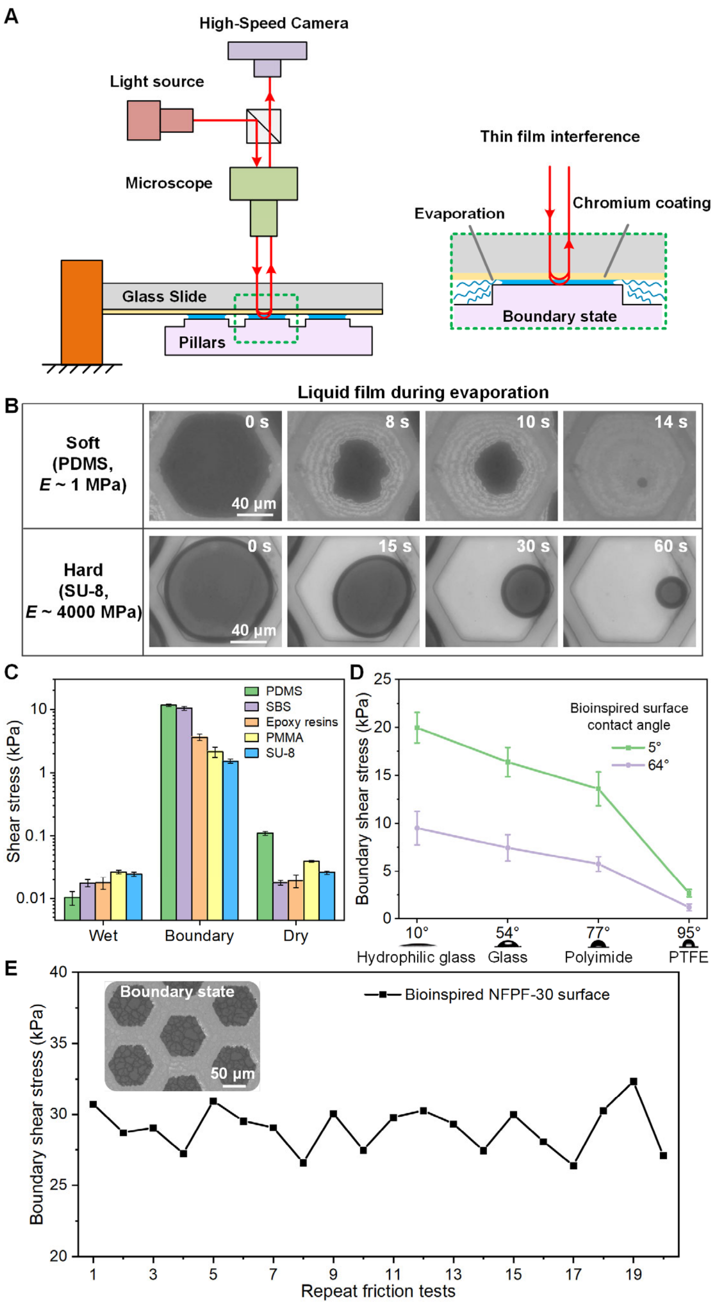

Fig. S7. *In-situ* characterization of interfacial liquid film behaviors on BP during the evaporation process and the influence of pillar material properties on the shear stress of BP surfaces. (**A**) Schematic illustration of observing interfacial liquid film on bioinspired surfaces during the evaporation process. (**B**) Characterization of pillar deformation induced by liquid bridge on soft and hard pillar surfaces during the interfacial liquid evaporation. (**C**) The wet friction performance of bioinspired BP surfaces fabricated with various materials. (**D**) The boundary friction of bioinspired BP surfaces with different water contact angles on various substrates. (**E**) The boundary friction of bioinspired NFPF-30 surfaces during 20 times friction tests.

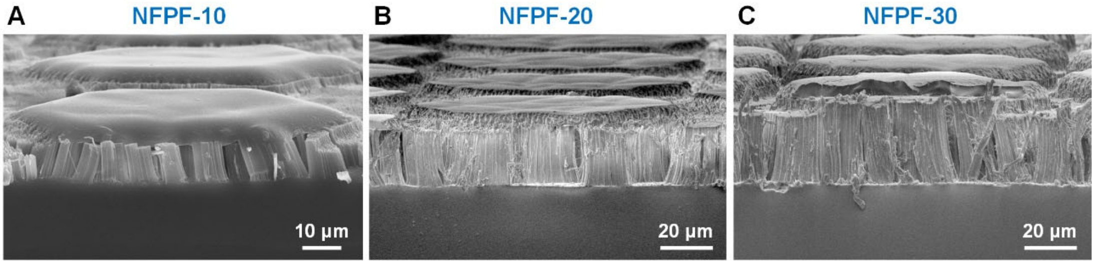

Fig. S8. Sectional view of fabricated NFPFs with different fiber heights. (**A, B, C**) SEM images of NFPFs with fiber heights of 10, 20, and 30 μm, respectively.

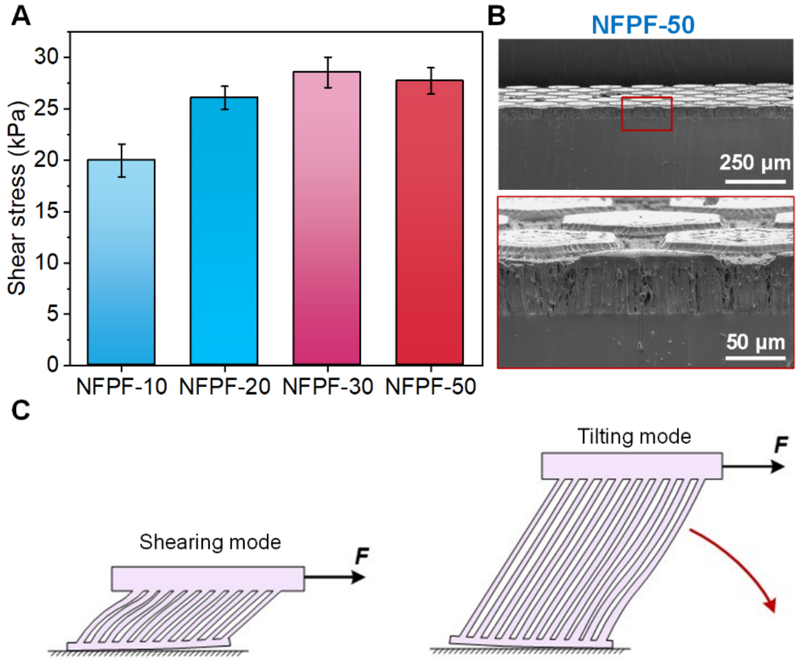

Fig. S9. The wet friction performance of bioinspired NFPF surfaces with different fiber heights. (**A**) Boundary frictional shear stress for NFPF surfaces with different fiber heights. (**B**) SEM images of NFPFs with the fiber height of 50 μm. (**C**) Schematic diagram of distinct deformation modes of NFPFs with different aspect ratios.

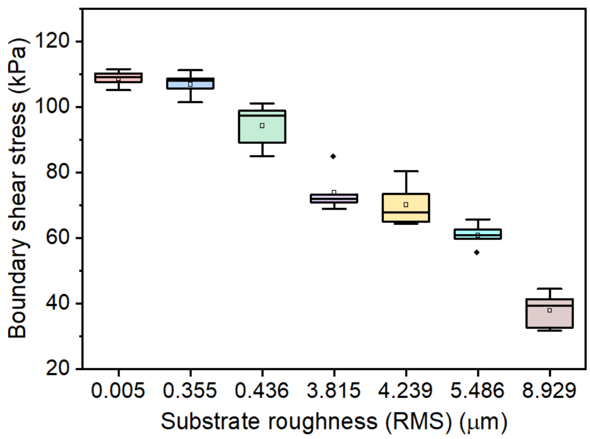

Fig. S10. The boundary shear stress of the Chinese bush cricket pad on different roughness substrates. All friction tests are measured with a normal load of ~1 kPa and a contact area of ~1 mm^2.

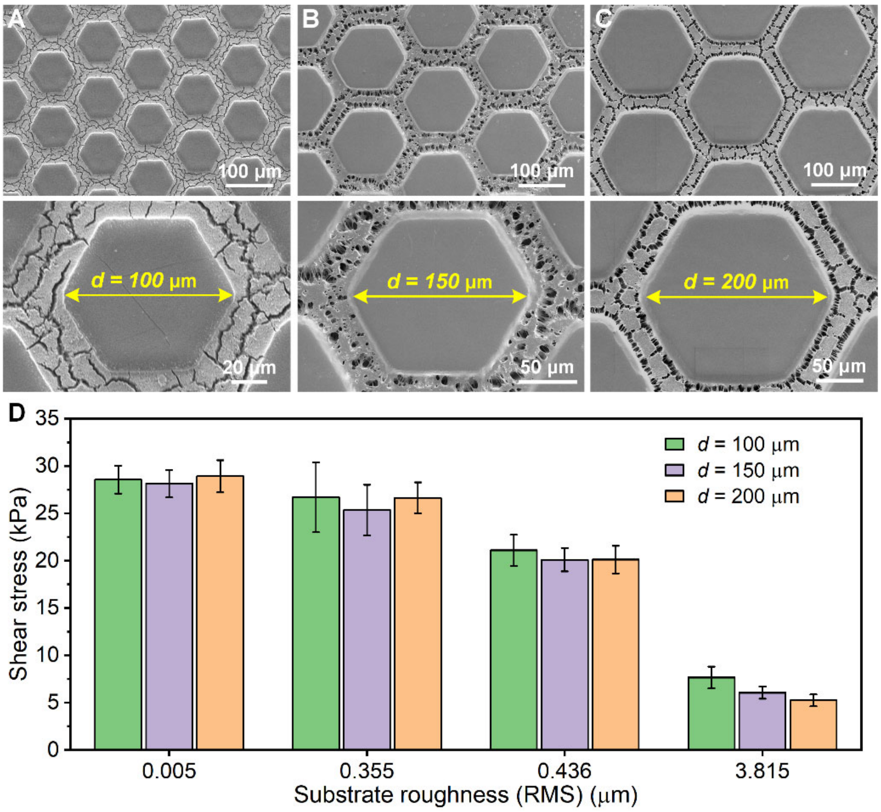

Fig. S11. SEM images of NFPF-30 with different pillar diameters and their friction performance on various roughness substrates during the boundary friction. (**A, B, C**) SEM images of NFPF-30 with pillar diameters of 100, 150, and 200 µm, respectively. (**D**) The shear stress of these NFPF-30 surfaces on substrates with various roughness in the boundary friction.

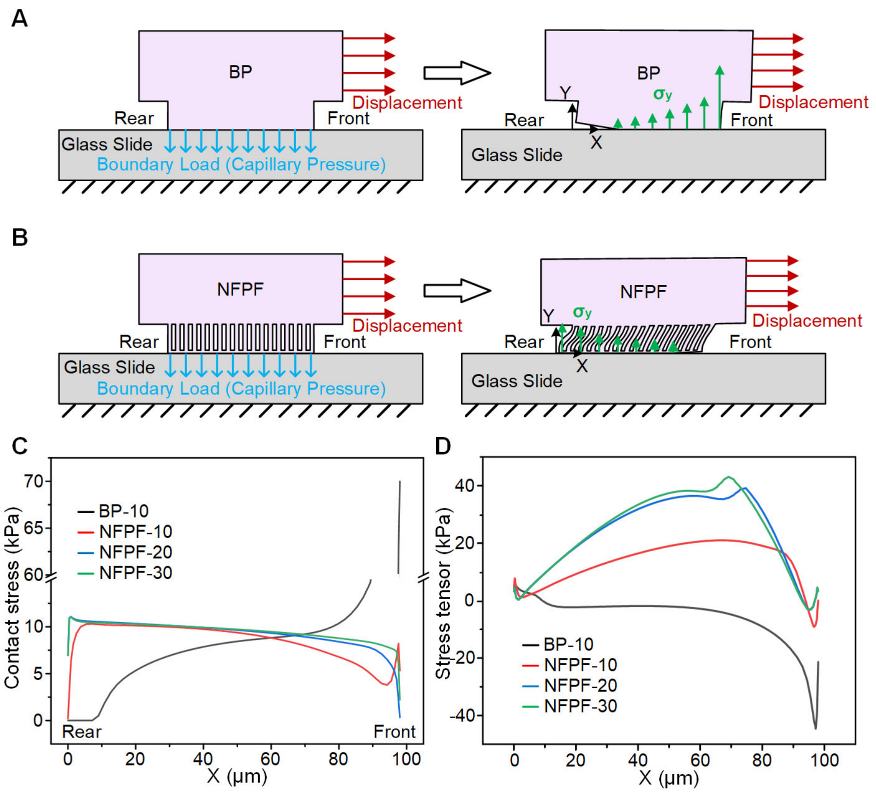

Fig. S12. Schematic diagram of setting up the finite element analysis (FEA) model and simulation results. (**A**) The FEA model for BP. (**B**) The FEA model for NFPF. (**C**) The contact stress distribution throughout the contact interface of different pillars. (**D**) The stress tensor distribution at the contact interface of different pillars.

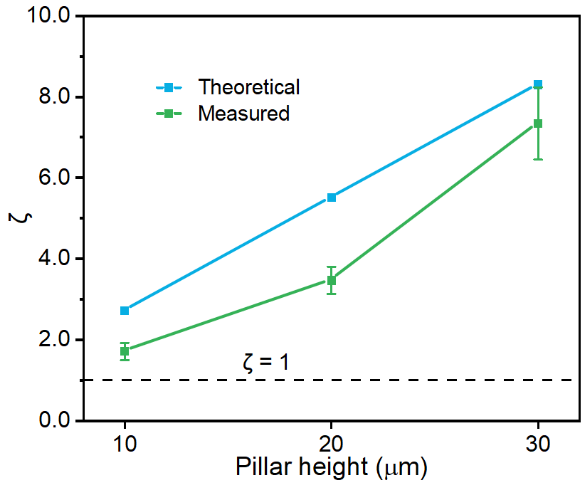

Fig. S13. Theoretical and experimental results of the ratio of the maximum lateral force on the NFPF to that on the BP. The ratio ζ of theoretical predictions and experimental measurements for bioinspired NFPF surfaces and BP surfaces in different heights.

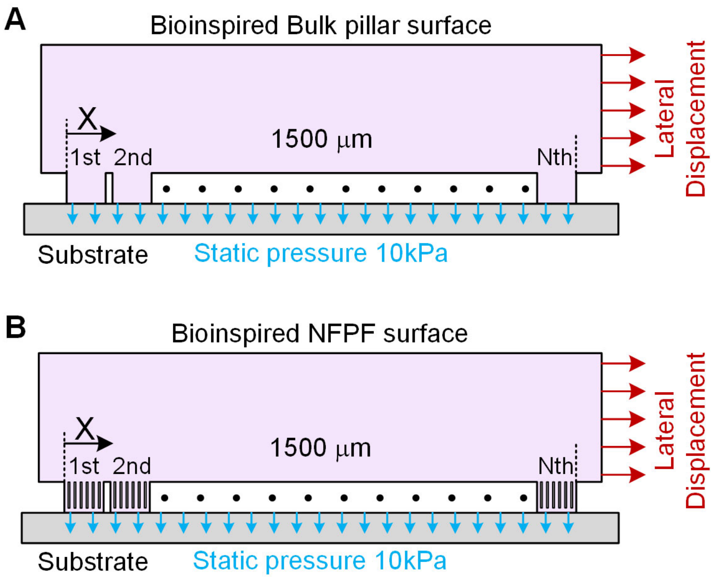

Fig. S14. FEA simulation for bioinspired pillar array surfaces sliding on a substrate with a different number of pillars. (**A**) FEA setup for bioinspired BP array surfaces. (**B**) FEA setup for bioinspired NFPF array surfaces. The lateral displacement is applied at one side of the bioinspired surface, and the downward static pressure is applied on each pillar to simulate the capillary pressure. A friction pair is set between the interface.

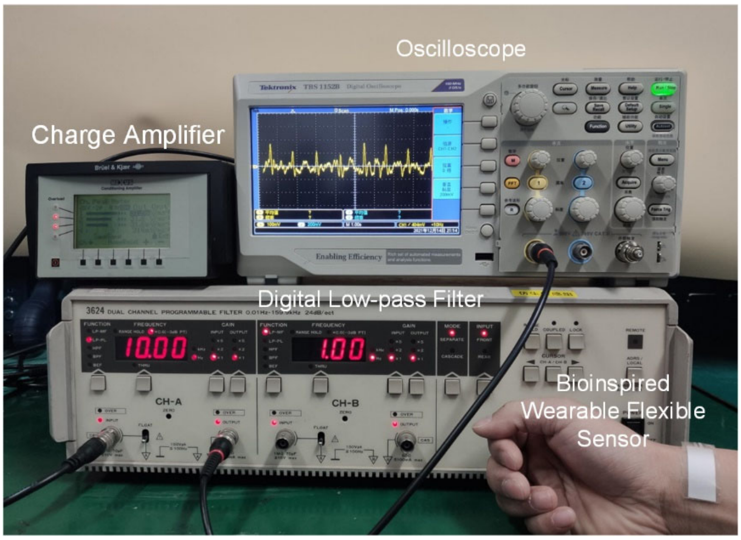

Fig. S15. Test equipment setup for bioinspired wearable flexible sensors. The electrical signals generated by the pulse vibration are processed by a charger amplifier and a digital low-pass filter, then displayed by an oscilloscope. Different volumes of water are added to the skin surface with a pipette to simulate different states of sweating.

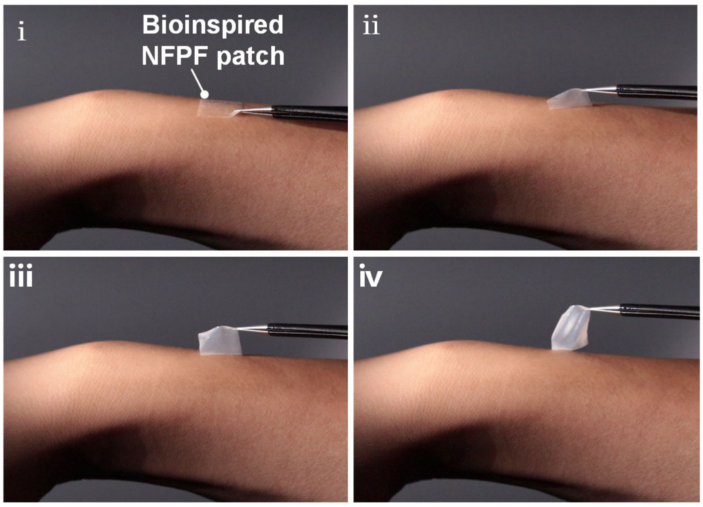

Fig. S16. The peeling process of bioinspired NFPF patch on volunteer's forearm. This bioinspired NFPF patch can be easily peeled off from the skin, and cause no pain to the skin like vacuum suckers or tissue adhesives do.

Table S1. **Structural parameters of NFPF surfaces and numerical results of the ratio ζ between the theoretical maximum friction on the NFPF and BP.**

Structural parameters						Theoretical value
L (μm)	h (μm)	d (μm)	h^s (mm)	ϕ (nm)	w (nm)	ζ
100	10	100	10	400	50	2.7
100	20	100	10	400	50	5.5
100	30	100	10	400	50	8.3

Captions for Movies:

Movie S1.
Characterization of the liquid film behavior on the Chinese bush cricket pad in friction.

Movie S2.
Characterization of the liquid film behavior and structural deformation on BP in friction.

Movie S3.
Characterization of the liquid film behavior and structural deformation on NFPF in friction.

Movie S4.
Characterization of the liquid film behavior and structural deformation on PSAN in friction.

Movie S5.
Flowing water resistance test for smooth and bioinspired surface patches.
